# Chlorhexidine and doxycycline gel versus chip as adjuncts in oral hygiene among Indians

**DOI:** 10.6026/973206300191342

**Published:** 2023-12-31

**Authors:** Ankur Singh Rajpoot, Hiroj Bagde, Sweety Thumar, Naina Pattnaik, Nairita Saha, Kanishq Patel, Dhaval Niranjan Mehta, Bibhukesh Panigrahi

**Affiliations:** 1 Department of Periodontology, RKDF Dental College and Research centre, Bhopal, M.P., India; 2 Department of Periodontology, Chhattisgarh Dental College and Research Institute, Rajnandgaon, Chhattisgarh, India; 3 Associate Professor, Department of Endodontics and Conservative dentistry, Karnavati School of Dentistry, Karnavati University, Gandhinagar, Gujarat, India; 4 Department of Periodontology, Kalinga Institute of Dental Sciences, KIIT Deemed to be University, Bhubaneswar, Odisha, India; 5 Department of Oral Medicine and Radiology, Burdwan Dental College & Hospital, Burdwan, West Bengal, India; 6 AMC dental College, Khokhra, Ahmedabad ,Gujarat,India, India; 7 Department of Oral Medicine and Radiology, Narsinbhai Patel Dental college and Hospital, Sankalchand Patel University, Visnagar, Gujarat, India; 8 Department of Periodontology, Awadh Dental & Hospital, Jamshedpur, Jharkhand, India

**Keywords:** Chlorhexidine, doxycycline, gel, chip, oral hygiene

## Abstract

The effectiveness of newly released medications such as Chlorhexidine (CHX) chip, Doxycycline hyclate (DH) chip, CHX gel, DH chip as adjunct to scaling and root planing in the treatment of chronic periodontitis is important. 90 adult Indian patients with moderate chronic periodontitis were enlisted. It was observed that reduction in periodontal pocket depth (PPD) and increase in clinical attachment level (CAL) was seen in patients in CHX group as compared to DH treated study participants. It was observed that CHX and DH in gel form were more effective in improving periodontal health as compared to CHX and DH in chip form in this group of subjects.

## Background:

A gradual deterioration of attachment and the development of a periodontal pocket are the outcomes of chronic periodontitis [1-2]. The pathologic result of bacterial and inflammatory-mediated damage of collagenous connective tissues as well as alveolar bone is the procedure of periodontal pocket formation [30- 4]. However, since the pathogenic bacteria are located in gingival tissues or other places that are unreachable to periodontal devices, treatment with mechanical devices may not be able to completely eradicate them [4-5]. Because chemical aids could make up for technological shortcomings and stop very early microbial colonization, the use of multiple antimicrobial agents began to gain traction [6-7]. This would ultimately guarantee an optimal opportunity for clinical advancements. There are two possible delivery routes for these chemical substances to enter the periodontal pocket: systemic and local [8-9]. The application of external antibacterial medications for the management of periodontitis is currently the subject of much research due to the potential negative consequences of systemic administration of antibiotics, including super infections, resistant strains and sensitivity [10-11]. Several drugs have been employed as mono-therapy or in conjunction with the root planing and scaling (SRP) procedure to stop the advancement of periodontal diseases [12- 14]. They include metronidazole, chlorhexidine, enzymes, minocycline, quaternary ammonium compounds, doxycycline and tetracycline and others [15-16]. These have been applied topically in their purest form by being incorporated into gels, chewing gum, films, dentifrices, ointments, hollow fibres and acrylic strips so forth [17-18]. It is evident that accurate strategies for providing a perpetuated and sufficient quantity of the drug's active ingredient within the periodontal pocket are necessary for regional antimicrobial medication to be clinically effective [19- 20]. Topical antiseptics have proven to be beneficial for managing gingivitis caused by plaque. Among these, chlorhexidine (CHX) is still one of the most potent antimicrobials that has been documented to date and is not known to exhibit significant antibiotic resistance among oral microbes [21-22].

However, because the drug could not be kept in the periodontal pocket for long enough to reach physiologically important concentrations, subgingival irrigation with CHX gels or solution proved to be ineffective in treating periodontitis [23- 24]. For a variety of reasons, including inadequate penetration by mouth rinses, quick irrigation solution dissipation, and relatively low localized concentrations attainable with high systemic doses of antibiotics, it is challenging to maintain the efficient antibacterial quantities in periodontal pockets for a long enough duration [25- 26].

Slow-release products were created as a result. There are two subtypes: "controlled delivery devices" (CDDs), which release the active ingredient over a longer duration of time, and "sustained release devices," which discharge the medication for no more than 24 hours [20-24]. A successful suppression of periodontal microorganisms necessitates the administration of an intrinsically efficient antimicrobial agent [21- 23]. When these substances arrive at the periodontal pocket, which is the location of action, they retain the low effective concentration long enough to have the intended targeted therapeutic effect [20- 21]. Doxycycline hyclate (DH) was among the first few antibiotics to be examined, and several periodontal clinical trials were conducted to assess its effectiveness [22,26]. Many local methods of administration have been developed for placing doxycycline hyclate into periodontal pockets. These consist of decomposable chip, ethyl cellulose fibers, ethylene vinyl acetate copolymer fibers, collagen preparations, acrylic strips and hollow fibers [25-26. Therefore, it is of interest to document the effectiveness of newly released medications such as chlorhexidine (CHX) chip, doxycycline hyclate (DH) chip, CHX gel, DH chip as adjunct to scaling and root planing in the treatment of chronic periodontitis.

## Methods and Materials:

The design of this clinical trial called for a three-month, prospective, single-center, randomized, controlled split mouth study. The study was registered in clinical trials of India website.

## Patient Sample:

Ninety adult patients with chronic periodontitis that was moderately advanced were enlisted.

## Qualifications for Inclusion:

Subjects in this study, both male and female, aged 25 to 75 years, and were enrolled if they met the following criteria:

[1] Recurrent or moderate-to-severe periodontitis that has not been treated with periodontal surgery for at least the previous 24 months.

[2] At least three teeth that bleed on probing during the first visit, with a probing pocket depth (PPD) of five to eight mm, evenly spaced throughout the mouth serving as isolated units for testing (i.e., at least one tooth apart) [11].

[3] Signed a voluntary informed consent form.

[4] Completed the health history questionnaire satisfactorily.

## Exclusion criteria:

Participants were disqualified if they

[1] Previously experienced oral candidiasis

[2] Experiencing allergies to chlorhexidine, doxycycline hyclate, or other tetracyclines

[3] Lactating or pregnant woman

[4] Teeth due to potential endodontic/periodontal issues

[5] Had subgingival instrumentation (SRP) inserted less than two months before the baseline assessment

## Interventions:

Based on split mouth design, five sites were chosen for each subject and randomly assigned to receive one of the five treatments. The teeth were in separate quadrants; however, the gel was positioned one tooth apart in instances where a couple of teeth in the same quadrant needed to be treated.

10% Doxycycline hyclate gel + SRP (DH (G)+SRP)

Xanthan based chlorhexidine gel + SRP (CHX (G)+SRP)

Doxycycline Hyclate chip + SRP (DHC (C) +SRP)

Chlorhexidine Chip +SRP (CHX (C) + SRP

SRP alone

## Clinical analysis:

The subjects underwent baseline, one-month, and three-month evaluations. The following variables were included in clinical examinations:

[1] The plaque index (PI)

[2] Gingival Index (GI)

[3] Probing pocket depth (PPD): measured to the nearest whole milli-metre, this is the distance, using a UNC no. 15 manual probe, between the gingival margin and the bottom of the probe able pocket.

[4] Clinical attachment level (CAL): the separation between the base of the pocket and the cemento-enamel junction (CEJ).

[5] Gingival margin location (GM): using a periodontal probe, determine the distance between the gingival margin and the CEJ or another well-defined landmark (acrylic stent) on the tooth.

CAL = PPD - GM is the formula used to calculate CAL. A reference splint provided the vertical relative attachment levels and indicated the probing location through notches. 14 Four sites surrounding each tooth (mid palatal/lingual, disto-buccal, mid-buccal and mesio-buccal) were used for the measurements. Alginate impressions were obtained at baseline, and occlusal acrylic stents were made to measure the attachment levels. Additionally, subjects were questioned about any adverse events that occurred and the use of concurrent medications and treatments at each visit.

## Procedures for treatment:

A full mouth supra- and subgingival SRP was administered to each subject using curettes and an ultrasonic scaler. The subjects were carefully instructed in self-maintained oral hygiene practices, which included brushing twice a day with a soft toothbrush and regular toothpaste containing fluoride, using the modified Bass brushing technique, and cleaning between teeth once a day with dental floss or interdental brushes. Throughout the study period, using antimicrobial mouthrinses was prohibited. For seven days, the subjects were instructed not to perform mechanical oral hygiene procedures (such as brushing or flossing their teeth) on any treated areas. At every recall visit, the patient's level of oral hygiene was assessed, and when necessary, more instructions were provided.

## Statistical analysis:

Statistical Package for Social Sciences (SPSS) version 2021 was used for statistical analysis. The mean change in attachment level and the average change in pocket probing depth were the main efficacy endpoints of the current trial. A measurement of the entire mouth was made. The subject mean, not just the sites, served as the foundation for the statistical analysis for all parameters. All subjects' values were averaged. Using the Paired Sample "t" test, the efficacy results for treated sites that met the requirements for both CAL and PPD were statistically analyzed.

## Results:

The reduction in PPD between baseline and one month follow up was 2.0±0.39mm in DH (G) ± SRP subgroup, while it was 2.73±0.45mm when evaluated between baseline and three months. Similarly, there was reduction of 0.73±0.06mm in PPD when evaluated between one month and three months. It was observed that there was statistically meaningful reduction in PPD at one month and three month follow up in subcategory of DH(G)+SRP. It was observed that decrease in periodontal pocket depth as compared between baseline and one month follow up in CHX (G)+SRP subcategory was 1.77±0.42mm. When there was comparison between baseline and three month follow up then reduction was 2.76±0.40mm. The reduction was 1.00±0.02mm when analyzed between one month and three month follow up. The reduction in periodontal pocket depth at 1 month follow up, 3 month follow up was statistically meaningful in CHX (G)+SRP subcategory.

The PPD declined from 7.27±1.28mm at baseline to 6.27±0.89mm at one month follow up and subsequently reduced to 5.74±0.83mm at three month follow up in DH(C)+SRP subcategory. The reduction in PPD at one month follow up and three month follow up compared to baseline was statistically significant (p>0.05). The PPD in CHX (C)+ SRP reduced from 7.51±0.90mm at baseline to 6.73± 1.32mm at one month follow up and further got decreased to 5.74±1.30mm at three month follow up. The reduction in PPD at one month follow, three month follow up was significant statistically. In case of SRP alone subcategory, the decline in PPD was 0.61±0.39mm at one month follow up and 1.43±0.45mm at three month follow up as compared to baseline. The reduction in PPD was statistically significant (p<0.05).

It was observed that there was significant improvement in periodontal health in all types of interventions at one month follow up and three month follow up. It was however observed that low improvement was observed in SRP alone subgroup as compared to other interventions. It was also observed that reduction in PPD was high in CHX treated study participants as compared to DH treated study participants. When there was further analysis, then it was observed that CHX and DH in gel form were more effective in improving periodontal health as compared to CHX and DH in chip form (Table 1). It was statistically not significant (p>0.05).

There was improvement in CAL from 7.45±0.46mm at baseline to 6.34±0.63mm at one month follow up, and 5.21±0.49 mm at three month follow up in DH (G)+SRP. The increase in CAL at one month follow up, three month follow up in comparison to baseline was statistically vital. It was observed that increase in CAL as compared between baseline and one month follow up in CHX(G)+SRP subcategory was1.50±0.06. When there was comparison between baseline and three month follow up then elevation was 2.59± 0.34mm. The elevation was 1.09±0.20 when analyzed between one month and three month follow up. The increase in CAL at 1 month follow up, 3 month follow up was statistically meaningful in CHX (G) + SRP subcategory.

The CAL improved from 7.56 ± 0.46 mm at baseline to 6.49±0.63mm at one month follow up and subsequently increased to 5.87±0.49mm at three month follow up in DH(C) + SRP subcategory. The elevation in CAL at one month follow up and three month follow up compared to baseline was meaningful statistically. The CAL in CHX (C)+ SRP increased from 7.74±0.46mm at baseline to 6.64± 0.32 mm at one month follow up and further got increased to 5.94±0.12 mm at three month follow up. The elevation in CAL at one month follow, three month follow up was significant statistically. In case of SRP alone subcategory, the increase in CAL was 1.07±0.74 mm at one month follow up and 1.69±0.37 at three month follow up as compared to baseline. The increase in CAL was significant statistically. It was observed that there was significant improvement in clinical attachment level in all types of interventions at one month follow up and three month follow up. It was however observed that low improvement was observed in SRP alone subgroup as compared to other interventions. It was also observed that elevation in CAL was high in CHX treated study participants as compared to DH treated study participants. When there was further analysis, then it was observed that CHX and DH in gel form were more effective in improving clinical attachment level as compared to CHX and DH in chip form (Table 2). It was statistically not significant (p>0.05). It was further observed that there was decrease of ≥ 2mm in PPD in 84.44%,85.12%, 83.43%,84.11% and 67.33% sites in DH (G)+SRP, CHX (G)+SRP, DH(C)+SRP, CHX (C)+ SRP and SRP alone respectively (Table 3). The percentage of such sites was low in SRP alone. The percentage of sites with decrease of ≥ 2mm was greater in CHX and DH in gel form as compared to CHX and DH in chip form (Table 3). The percentage of sites with relative gain of ≥ 2mm and relative gain of 1mm was greater in CHX and DH in gel form as compared to CHX and DH in chip form (Table 4).

## Discussion:

It was observed that there was significant improvement in periodontal health in all types of interventions at one month follow up and three month follow up. It was however observed that low improvement was observed in SRP alone subgroup as compared to other interventions. It was also observed that reduction in PPD in was high in CHX treated study participants as compared to DH treated study participants. There were some studies conducted earlier like present study, which showed improvement in periodontal health and CAL on adding local drug delivery system consisting of chlorhexidine and doxycycline hyclate with SRP [18,19, 23,25]. However, some studies also showed no significant additional improvement in periodontal health and CAL in using such local drug delivery system. Some studies were in line with our study to show that CHX produced better results than DH [13-16, 23]. A study also showed that CHX and DH in gel form produced more reduction in PPD than in chip form [25].

In this study, a manual UNC #15 periodontal probe with a visual readout that was not force-controlled was used for all measurements. For controlled clinical trials, force-controlled automated probes were recommended to guarantee accurate measurements [4, 6,7,9]. Others, on the other hand, did not note a discernible improvement in PPD measurement reproducibility when using a force-controlled probe with a visual read-out to the closest 0.5 mm instead of a straightforward manual probe. A study found that manual probes had superior intra- and inter-individual reproducibility when compared to automated force-controlled probes (peri-probe, floridia probe) and simple (TPS) probes. Using a reference stent, all measurements were highly standardized. The occlusal stent reference improves measurement reproducibility and reliability [4,6]. It was observed that there was significant improvement in clinical attachment level in all types of interventions at one month follow up and three month follow up. It was however observed that low improvement was observed in SRP alone subgroup as compared to other interventions. It was also observed that elevation in CAL was high in CHX treated study participants as compared to DH treated study participants. When there was further analysis, then it was observed that CHX and DH in gel form were more effective in improving clinical attachment level as compared to CHX and DH in chip form.

For the duration of the study, the gingival and plaque indices both stayed satisfactory, indicating that patients followed the recommendations regarding oral hygiene. The thoroughness of SRP and the maintenance of good oral hygiene may be the cause of the decline in gingival and plaque scores. Following extensive SRP (alone), clinical improvements, a decrease in PPD, and an increase in CAL were observed. These data appear to be related to a decrease in inflammation brought on by changes in the subgingival bacteria [23-25]. It has recently been suggested that a scaling procedure may induce a local and systemic host response in addition to removing local etiological factors, which would help to eradicate local infection and encourage healing. In addition, a healing stage in which fresh epithelial attachment along with connective attachment will develop into a renewed periodontal support would follow the deliberate and/or unintentional removal of inflammatory tissue as well as pocket epithelium related to SRP [21-23]. There are studies that showed SRP alone also cause significant reduction in PPD and elevation of CAL provided the patient also maintain self-oral hygiene [20,22, 23].

The clinical decrease in PPD in the DH+SRP group, which received doxycycline treatment, was significant and correlated with a decrease in gingival tissue inflammation. The principal cause of the decrease in periodontal probe penetration depth following standard treatment has been associated with decrease in inflammation and healing in the connective tissue adjacent to the junction epithelium [24]. The extra ability of doxycycline to reduce tissue collagenase activity may contribute to the enhanced response [26]. The potential for doxycycline to attach onto the mineralized dental enamel and function as a short-term storage facility of an antimicrobial substance during an extended period may have contributed to the apparent greater closure of pockets [23,24]. The bactericidal concentrations attained at the chosen sites on day 1 of the chlorhexidine treatment group, CHX+SRP, are responsible for the PPD reduction. High concentration levels were sustained for the next two weeks. Therefore, in the absence of or after a reduction in the microbial load, improved healing may have happened at the test sites [15,16, 19]. The absence of bacterial encourage during the crucial early stage of healing after SRP may be the cause of the greater improvement in CAL in DH+SRP and CHX+SRP. The observations in this investigation are consistent with known data [19,21]. In DH+SRP and CHX+SRP, the proportion of sites exhibiting a pocket depth decline of less than 2 mm at three months was comparable. There were some other studies which showed observations different from our study which showed statistically better results with chlorhexidne especially in gel form as compared to chip form [19,20,23, 25].

## Conclusion:

The chlorhexidine and doxycycline in gel form as well as chip form can be used as adjunct to SRP in improving periodontal health.

## Figures and Tables

**Table 1:** PPD measurements (mean ± SD) in mm compared in different treatment protocols at different time intervals

	**DH (G)+SRP**	**CHX (G)+SRP**	**DH(C)+SRP**	**CHX (C)+ SRP**	**SRP alone**
Baseline	7.27±1.28	7.51±0.90	7.27±1.28	7.51±0.90	7.39±1.28
One month	5.27±0.89	5.74± 1.32	6.27±0.89	6.73± 1.32	6.78±0.89
Three month	4.54±0.83	4.74±1.30	5.74±0.83	5.74±1.30	5.96±0.83
**Comparison**					
Baseline vs One month	2.0±0.39	1.77±0.42	1.0±0.39	0.78±0.42	0. 61±0.39
t value	13.2	12.95	11.19	10.84	9.18
P value	0.001	0.001	0.001	0.001	0.001
Baseline versus three month	2.73±0.45	2.76±0.40	1.73±0.45	1.76±0.40	1.43±0.45
t value	12.2	13.22	10.15	11.14	8.15
P value	0.001	0.001	0.001	0.001	0.001
One month versus three month	0.73±0.06	1.00±0.02	0.53±0.06	0.99±0.02	0.82±0.06
t value	5.28	7.13	3.14	5.2	3.08
P value	0.001*	0.001*	0.001*	0.001*	0.001*
*indicates statistically significant results

**Table 2:** CAL measurements in mm (Mean ± SD) at different time period in different treatment modalities

	**DH (G)+SRP**	**CHX (G)+SRP**	**DH(C)+SRP**	**CHX (C)+ SRP**	**SRP alone**
Baseline	7.45±0.46	7.63±0.46	7.56±0.46	7.74±0.46	7.76±0.46
One month	6.34±0.63	6.13± 0.32	6.49±0.63	6.64± 0.32	6.75±0.63
Three month	5.21±0.49	5.04±0.12	5.87±0.49	5.94±0.12	5.98±0.49
**Comparison**					
Baseline vs One month	1.11±0.23	1.50±0.06	1.07±0.74	1.10±0.26	1.07±0.74
t value	11.35	12.12	10.24	11.1	10.24
P value	0.001	0.001	0.001	0.001	0.001
Baseline versus three month	2.24±0.12	2.59± 0.34	1.69±0.37	1.80±0.34	1.69±0.37
t value	12.26	13.36	12.26	13.36	12.26
P value	0.001	0.001	0.001	0.001	0.001
One month versus three month	1.13±0.14	1.09±0.20	0.62±0.14	0.71±0.12	0.62±0.14
t value	5.14	6.21	5.14	6.21	5.14
P value	0.001	0.001	0.001	0.001	0.001

**Table 3:** Percentage of sites showing PPD change in different groups from baseline to 3 months

	**Decrease of 1mm**	**Decrease ≥ 2mm**	**No change**
DH (G)+SRP	11.12%	84.44%	4.44%
CHX (G)+SRP	12.24%	85.12%	2.64%
DH(C)+SRP	10.14%	83.43%	6.43%
CHX (C)+ SRP	11.26%	84.11%	4.63%
SRP alone	21.00%	67.33%	11.67%

**Table 4:** Percentage of sites showing relative CAL in different groups from baseline to 3 months

	**Relative gain of 1mm**	**Relative gain ≥ 2mm**	**No change**
DH (G)+SRP	44.12%	54.34%	1.54%
CHX (G)+SRP	27.21%	65.44%	7.35%
DH(C)+SRP	46.14%	51.33%	1.53%
CHX (C)+ SRP	29.21%	64.44%	6.35%
SRP alone	54.57%	17.34%	28.09
